# Pneumopericardium causing pericardial tamponade

**DOI:** 10.1002/ccr3.3233

**Published:** 2020-08-10

**Authors:** Ryan J. Fink

**Affiliations:** ^1^ Department of Anesthesiology and Perioperative Medicine Oregon Health & Science University Portland Oregon USA

**Keywords:** anesthesia, cardiovascular disorders, critical care medicine

## Abstract

Pneumopericardium can be severe enough to cause pericardial tamponade physiology. These patients can be hemodynamically unstable and require pericardial drainage.

## Graphical abstract

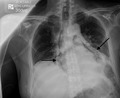

## 1 CASE AND IMAGE DESCRIPTION

Pneumopericardium, or gas in the pericardial sac, is a rare condition resulting from complications from thoracic trauma, malignancies, and mechanical ventilation, or is iatrogenic.^1^ It typically presents with chest pain, pericarditis, or tamponade physiology.^1^ The patient presented had a repeat Nissen fundoplication 6 months prior to presentation. He presented with shortness of breath, chest pain, hypotension, tachycardia, and a pulsus paradoxus of 20 mm Hg, consistent with pericardial tamponade.

**FIGURE 1:**
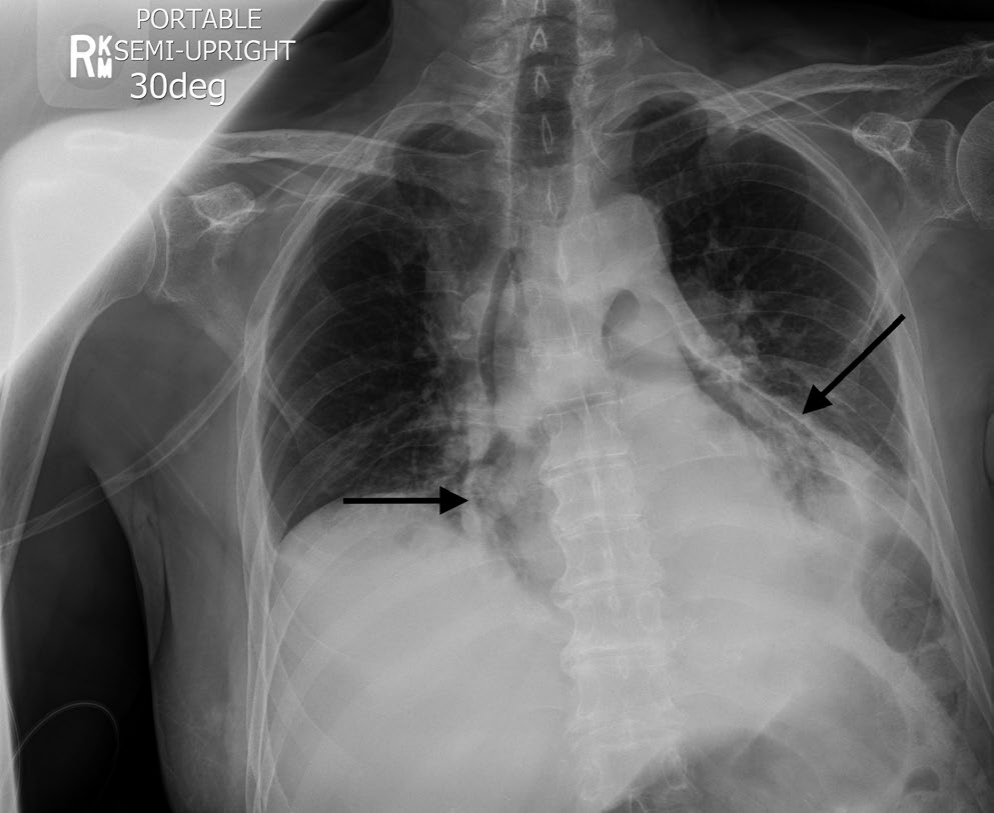
Chest radiograph which shows a band of gas curving around the cardiac chambers, with a sharp demarcation by the pericardium (black arrows)

**FIGURE 2:**
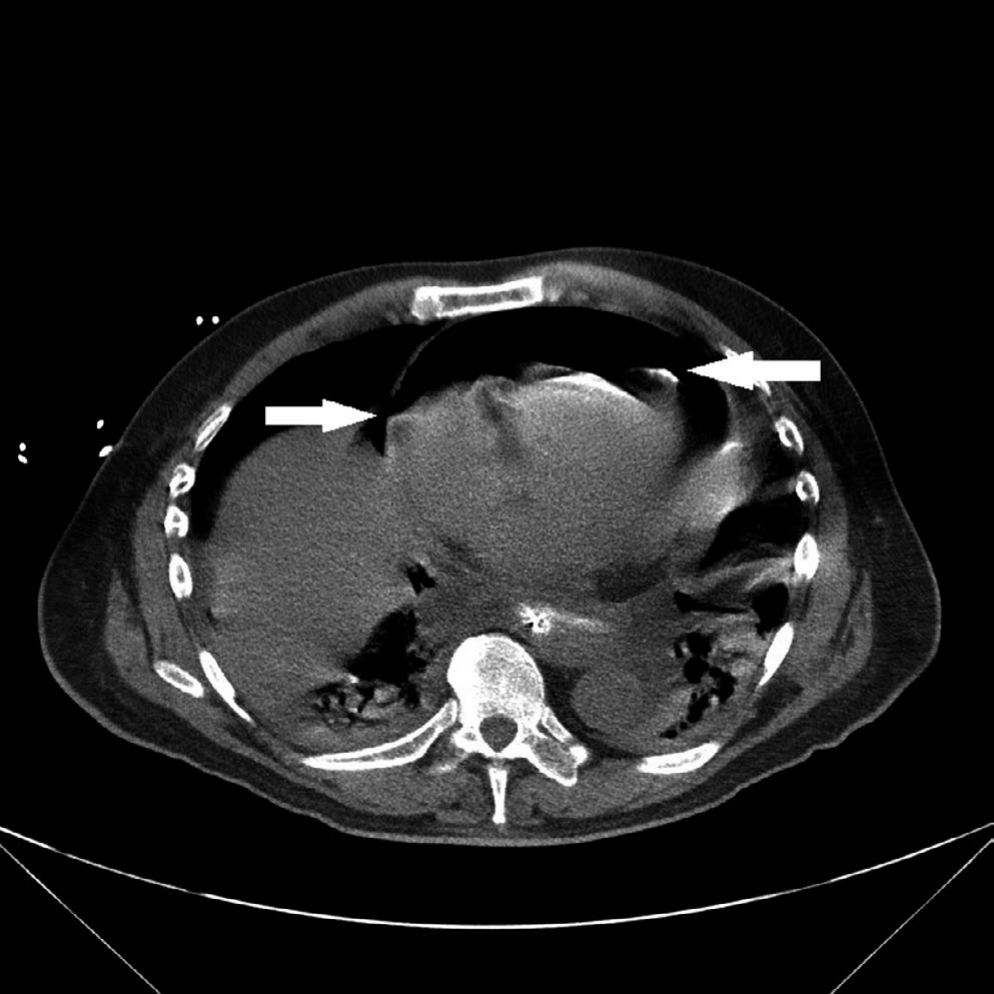
Computed tomography scan which shows dependent air‐fluid levels in the pericardial sac (white arrows)

These images are typical of pneumopericardium. The chest radiograph (Figure 1) shows a band of gas curving around the cardiac chambers, with a sharp demarcation by the pericardium (black arrows). The computed tomography scan (Figure 2) shows dependent air‐fluid levels, in the pericardial sac (white arrows). It may be challenging to distinguish pneumopericardium from pneumomediastinum, but the latter usually presents as multiple streaks of radiolucency that are not confined to the cardiac chambers.^2^ Diagnosis of pneumopericardium, especially pericardial tamponade, is especially important to diagnose preoperatively, as anesthetic induction can be life‐threatening.

This patient required emergent pericardial window, with air and gastric contents found in the pericardial sac. Esophagogastroduodenoscopy confirmed gastropericardial fistula, a complication from the surgical repair of a recurrent hiatal hernia. He eventually received an esophagogastric anastomosis for gastrointestinal tract continuity.

## CONFLICT OF INTEREST

None declared.

## AUTHOR CONTRIBUTIONS

RJF: collected the images, obtained IRB approval, and wrote the text.

## ETHICAL APPROVAL

This project was reviewed by the IRB.

## References

[1] Gołota JJ , Orłowski T , Iwanowicz K , Snarska J . Air tamponade of the heart. Kardiochir Torakochirurgia Pol. 2016;13:150‐153. 27516791 10.5114/kitp.2016.61052 PMC4971273

[2] Bejvan SM , Godwin JD . Pneumomediastinum: old signs and new signs. AJR Am J Roentgenol. 1996;166:1041‐1048. 8615238 10.2214/ajr.166.5.8615238

